# Exploring recruitment barriers and facilitators in early cancer detection trials: the use of pre-trial focus groups

**DOI:** 10.1186/1745-6215-15-98

**Published:** 2014-03-29

**Authors:** Roshan das Nair, Kate Skellington Orr, Kavita Vedhara, Denise Kendrick

**Affiliations:** 1 Division of Rehabilitation & Ageing, University of Nottingham, Queens Medical Centre, B98, B Floor, Nottingham NG7 2UH, UK; 2 KSO Research Limited, Radleigh House, 1 Golf Road, Glasgow G76 7HU, UK; 3 Division of Primary Care, University of Nottingham, Floor 13, Tower Building, University Park, Nottingham NG7 2RD, UK

**Keywords:** Screening, Cancer, Focus groups, Qualitative, Recruitment, Pretrial

## Abstract

**Background:**

Recruiting to randomized controlled trials is fraught with challenges; with less than one third recruiting to their original target. In preparation for a trial evaluating the effectiveness of a blood test to screen for lung cancer (the ECLS trial), we conducted a qualitative study to explore the potential barriers and facilitators that would impact recruitment.

**Methods:**

Thirty two people recruited from community settings took part in four focus groups in Glasgow and Dundee (UK). Thematic analysis was used to code the data and develop themes.

**Results:**

Three sub-themes were developed under the larger theme of recruitment strategies. The first of these themes, recruitment options, considered that participants largely felt that the invitation to participate letter should come from GPs, with postal reminders and face-to-face reminders during primary care contacts. The second theme dealt with understanding randomization and issues related to the control group (where bloods were taken but not tested). Some participants struggled with the concept or need for randomization, or for the need for a control group. Some reported that they would not consider taking part if allocated to the control group, but others were motivated to take part even if allocated to the control group by altruism. The final theme considered perceived barriers to participation and included practical barriers (such as flexible appointments and reimbursement of travel expenses) and psychosocial barriers (such as feeling stigmatized because of their smoking status and worries about being coerced into stopping smoking).

**Conclusions:**

Focus groups provided useful information which resulted in numerous changes to proposed trial documentation and processes. This was in order to address participants information needs, improve comprehension of the trial documentation, enhance facilitators and remove barriers to participation. The modifications made in light of these findings may enhance trial recruitment and future trials may wish to consider use of pretrial focus groups.

## Background

Of all the Medical Research Council and Health Technology Assessment Programme randomised controlled trials (RCTs) conducted between 1994 and 2003 (n = 114), less than one third recruited their original target within the time originally specified [1]. Bower, Wilson and Mathers [2] also found that less than a third of UK primary care trials recruited to their original timescale. Therefore, recruitment to trials is clearly one of the biggest challenges for trialists, and although often dealing with larger participant pools, recruitment to primary care trials is no exception [2,3]. The consequences of poor recruitment are: premature closure of trials, trials that are underpowered to answer the main research questions (and the dangers associated with this [4]), wasting of resources and the end-users of research (patients and clinicians) not benefiting from the intended outcomes of the trial.

In a systematic review, Ross *et al.*[5] documented the barriers to participation in RCTs. They identified both clinician barriers and patient barriers. The latter included: additional demands of the trial in terms of additional procedures and appointments, patient preferences for particular treatments, worry caused by uncertainty of the choice of treatments or random allocation, and concerns about amount and format of providing information and obtaining consent. Another systematic review on barriers to participating in cancer trials [6] identified system-related and organizational barriers, trial design barriers and individual health-care provider barriers. They did not specifically consider participant-related barriers in terms of how participants’ understanding of the trial might attract or dissuade them from participation. However, the authors do suggest a checklist to consider from a patient perspective, which includes the following questions: ‘What key information needs to be given to enable patients to feel more comfortable with the uncertainties involved in the trial and the concept of clinical equipoise?’, ‘How might the timing of the request to participate in the trial be sensitively addressed?’, ‘How might practical barriers such as cost to patients, transport and time commitments be addressed?’, and ‘How might the benefits of the trial be explained to patients?’. They also recommended that trialists prospectively identify the issues relevant to a particular trial using their findings as a starting point. Although this review was specific to cancer, it mostly focused on cancer treatment trials or hypothetical scenarios, and not cancer screening trials. The authors acknowledge that their findings need to be treated with caution due to a number of threats to the internal and external validity of the included studies. This review also points to the need for more research on barriers and moderators to trial participation for cancer screening trials. Furthermore, Weller and Campbell [7] call for *informed uptake* whereby participants make informed choices and are aware of all the risks and benefits of participation. These authors and the systematic review by Baron *et al.*[8] conclude that more research is required to evaluate the effectiveness of strategies that improve uptake to various cancer screening programs.

A Cochrane Review [9] considered the effectiveness of various strategies to improve recruitment to RCTs, and recommended using certain strategies (for example, telephone reminders). This review, however, did not include any studies that evaluated the effectiveness of using user tested trial material (including participant information leaflets [PILs] and consent forms) compared to standard researcher/industry produced material on recruitment rates. Knapp *et al.*[10] made such a comparison for a trial in acute myeloid leukemia (AML16). The authors concluded that user vetted trial material and procedures may have a positive impact on recruitment rates.

Information about RCTs can be confusing for participants [11], especially where consent is obtained following postal information (such as in PILs) as opposed to information provided face-to-face [12]. The ethical underpinning of RCTs assumes participants understand the concepts of randomization and equipoise, and can therefore give informed consent [13]. Screening trials have additional complexities as they typically seek to recruit people without disease symptoms, the consequences of participation are less clear than in treatment trials as the results may warrant further tests, and participants also need to understand the meaning of screening results.

In preparation for the first UK trial evaluating the effectiveness of screening using a blood test for the early detection of lung cancer (the ECLS trial, trial registration number: NCT01925625), we conducted a qualitative investigation designed to facilitate recruitment to the trial. This involved, but was not limited to, eliciting responses from members of the public representative of the target patient group to some of the questions posed by Fayter *et al.*[6]. The aims of the ECLS trial are to assess the effectiveness and cost effectiveness of the EarlyCDT-Lung test in increasing early stage lung cancer detection, thereby reducing the rate of late stage presentation and to assess effectiveness in reducing adverse outcomes, including potential psychological and behavioral consequences. The trial is set in general practices, predominately within the lowest quintile of deprivation measured using the Scottish Index of Multiple Deprivation, in NHS Tayside and NHS Greater Glasgow & Clyde. Adults aged 50 to 75 will be eligible to participate if they are current or former cigarette smokers with at least 20 pack-years, or have a history of cigarette smoking less than 20 pack-years plus a family history (mother, father, brother, sister) of lung cancer and who are healthy enough to undergo pulmonary resection or stereotactic radiotherapy. Participants will be invited by postal invitation from their general practitioner. Other members of the community living in these areas who hear about the trial though a range of publicity and meet the eligibility criteria, but are not registered at participating practices, will also be eligible to participate. The trial has two arms; the intervention arm, in which participants receive the EarlyCDT-Lung test, and the control arm, in which participants have blood taken which would be used for future cancer related research but not tested for lung cancer. The decision to take a blood sample in the control arm was based on research which suggests altruism can be a motivating factor for people to participate in research [5,14] and that participants may prefer to make some contribution to cancer research above that of completing questionnaires. Those with a positive EarlyCDT-Lung test will be followed up by imaging studies including chest x-rays and CT scans. The primary outcome measure is the difference at 24 months after randomization between the rates of patients with stage 3, 4 or unclassified lung cancer at diagnosis in the intervention arm, and those in the control arm. As the ECLS trial recruits older smokers and ex-smokers in economically disadvantaged areas, characterized by relatively low educational attainment and poor engagement with health services [15], we recognized that this may offer unique challenges to recruitment. For this reason participants’ views on the following issues were elicited: issues likely to influence recruitment into the trial and willingness to be randomized (including recruitment strategies, understanding of risk information, clinical equipoise and randomization); recruitment and study documentation (for example the invitation letter and PILs); factors which may facilitate and hinder trial participation; and understanding of aspects of the trial based on the PILs we developed.

Qualitative research is particularly helpful in getting an in depth appreciation of such views. In fact, it is perhaps their value in RCTs that has led researchers to develop standard operating procedures for clinical trial units that intend to use qualitative methods within traditional RCT designs [16]. Previous studies have successfully used qualitative research methods to improve recruitment to RCTs (for example The ProtecT [Prostate testing for cancer and Treatment], [17]). The Medical Research Council (MRC) has also identified the value of incorporating qualitative studies to RCTs (in mixed methods paradigms) [18].

## Methods

A focus group design was used for this study. Focus groups, rather than individual interviews, were considered because it allowed us to collect a large corpus of data within a short period of time from a large number of people. Furthermore, as Gibbs [19] suggests, the attitudes, feelings, and beliefs of participants that may be partially independent of a group (or its social setting) are more likely to be revealed through interaction between group members. Participants were recruited who met the inclusion criteria for the ECLS trial: age 50 to 75 years; current or former cigarette smokers with at least 20 pack-years, or a history of cigarette smoking plus family history of lung cancer; and living in the four catchment areas the main trial intends to recruit from, covering some of the most disadvantaged areas in Glasgow (Castlemilk and Darnley) and Dundee (Charleston and Douglas). We considered the focus groups provided patient and public involvement and did not seek other contributions from patients or the public to this stage of the pretrial work.

Recruitment took place in shopping centers and on streets close to other facilities such as community centers. Recruiters worked to broad quotas per group for age (50 to 59, 60 to 69, 70 to 75; quota minimum 3 per group), gender (quota: 5 male, 5 female), catchment area (10 per area), and working status (a diverse range fulfilling the following categories: full time (30 hours plus), part-time (8 to 29 hours), retired, house person, unemployed, student, long term sick, disability allowance), aiming to recruit 10 participants to each focus group. The focus groups were held during two afternoon and two evening sessions to allow people of different working status to attend (full time workers were more likely to be available to attend evening meetings). To maximize accessibility and familiarity of venues among participants, all groups were held in local community centers. We believed that this would enable us to get a diverse range of views from people who would potentially be eligible to take part in the trial. Groups were facilitated by one of the authors (KSO) and lasted approximately two hours. All participants received a £30 cash incentive for taking part, given at the end of the sessions.

A topic guide, based on previous literature and discussions with the researchers of the main trial, was developed for the focus group sessions (see Appendix). This covered a range of topics, including: likely willingness to take part in such a trial, barriers and facilitators to participation, understanding aspects of the trial (such as the concept of randomization, need for a control group, and so on), and views on various draft materials that had been developed for the main trial. Draft material given to the participants to comment on included: GP letter, PIL, and a summary sheet explaining the study in brief. We also displayed show cards with different recruitment options, a flowchart explaining the trial, and examples of leaflets used in other trials (to demonstrate different formats of conveying study information).

Written consent to participate was obtained from all participants. All sessions were digitally recorded and verbatim transcripts were produced to facilitate analysis. A combined inductive and deductive approach to thematic analysis was conducted [20]. All transcripts were read by two of the authors (RdN and KSO), and the core messages highlighted and extrapolated. These underwent an assimilation process so that similar responses were clustered together, which were then used to structure presentation of findings. Through discussion, we came to an agreement of the clustering of the data. We did not aim to achieve data saturation. This is a contested term in qualitative research and there are strong arguments against the use of data saturation as a quality indicator [21,22]. Transparency is considered more important than concepts such as saturation [21]. Therefore, we opted to follow Spencer *et al.*[23] to demonstrate transparency by ensuring sampling adequacy (in using a form of quota sampling) provided both depth and opportunities for transferability of findings (as reported in [21]). Furthermore, we explicitly provide excerpts from the data as participant quotes that exemplify the theme, so readers can see how the participant data maps onto our themes. For this study we considered theoretical sufficiency [24], which allowed us to build and work on constructs that emerged from the data. There was no member checking done. While member checking (or respondent validation) has been conducted in some studies, we followed the cautions of member checking raised by qualitative researchers such as Morse [25], Angen [26] and Sandelowski [27], who offer a comprehensive critical review of the use of member checks for establishing the validity of qualitative research.

The study was granted ethical approval from the Institute of Work, Health & Organisations at the University of Nottingham, UK.

## Results

### The sample

A total of 116 people were approached to take part. Of these, 40 (34%) agreed to participate, 15 were ineligible, and 61 declined. Of the 15 people who were ineligible, 11 did not meet the relevant smoking status criteria and four were under 50. Of the 61 people who declined to participate, 24 were not interested in taking part or gave no reason for refusal, 14 did not have the time, 8 were working at the time of the focus group, 7 had other responsibilities, 4 did not feel well enough to attend, 2 were not interested in stopping smoking and did not want to be advised about smoking, and 2 did not want to go out in the local area at night. Overall, there was little difference in nonparticipation by gender, with 37 women and 39 men declining, or ineligible to take part, with the exception of one site where it proved more difficult to recruit men than women. The average age of participants who agreed and declined to participate was 63 and 64 respectively. The groups did, however, differ somewhat in terms of working status, with the majority of those who agreed to participate being retired (47%), and the majority who declined being unemployed (49%).

Of the 40 people who agreed to participate, 32 (18 female, 14 male) attended one of the four focus groups. The mean age of participants was 63 years (range 50 to 75). The working status of the participants is described in Table 1.

**Table 1:** The working status of participants, by gender

**Working Status**	**Male**	**Female**	**All**
Full Time	2 (6%)	-	2 (6%)
Part Time	-	4 (13%)	4 (13%)
Retired	7 (22%)	8 (25%)	15 (47%)
House person	-	2 (6%)	2 (6%)
Unemployed	1 (3%)	4 (13%)	5 (16%)
Long Term Sick	2 (6%)	-	2 (6%)
Disability Allowance	2 (6%)	-	2 (6%)
**All**	**14 (44%)**	**18 (56%)**	**32 (100%)**

Of the 32 people who participated, almost all were current smokers (n = 31; 97%), with only one person stating that they had stopped smoking. The majority of participants reported smoking for 40 years or more (n = 28; 88%). Only four participants (12%) reported smoking for more than 20 years, but for less than 40 years. Almost all participants reported smoking one pack or more per day (n = 26; 81%), with only six (19%) reporting smoking less than one pack per day.

This paper presents our findings in relation to participant views regarding recruitment strategies only. Three themes were developed under the superordinate theme of recruitment strategies: recruitment options, understanding randomization and issues related to the control group, and perceived barriers and facilitators to participation. Each of these is considered below. To protect the anonymity of participants we present only their gender, age, and location.

### Recruitment options

A dedicated section of the focus group sessions explored the various recruitment options that could potentially be used for the main trial. Participants were asked to consider the following options: (A) sending a letter from GPs, with a free post envelope, asking patients to send back a reply slip to show if they are interested and providing contact details; (B) phoning patients who do not send back the reply slip to see whether they are interested (the letter from the GP would make it clear to people that they should contact the trial team if they do not want the trial team to contact them); (C) researchers handing out leaflets about the study to patients when they go to the GP surgery; (D) GP practice receptionists handing out leaflets to patients when they book in to see the GP or nurse, or when they collect a repeat prescription; or (E) GPs and nurses asking patients when they see them in consultations if they have not responded to study invite letters.

### The invitation to participate

The groups expressed quite different views with regards to the use of a GP letter as a means of recruiting into the study (option A). In Castlemilk (Glasgow, UK), there were no objections to this although some participants questioned whether GPs would really be on board for a study of this kind, and participants suggested that GPs were too remote and difficult to engage with:

‘Not through the GP. I wouldn’t bother contacting them and asking them to do it, ‘cause they’re hopeless.’ (Male 57, Castlemilk)

‘Cause sometimes, they [GPs] don’t even know what they’re doing. I don’t think that [health] center is up to date, and it takes you a week [to get an appointment], and when you get there, you’re feeling better.’ (Female 57, Castlemilk)

A further concern raised was as to whether the preparation and postage of letters would distract GPs from their routine activities. Despite this, the majority of participants in Glasgow seemed satisfied that the GP letter approach would work well, and most indicated that they would respond to such an invitation. In Charleston (Dundee, UK), however, some respondents expressed stronger reservations about whether the GP letter approach would work. People talked about letters being set aside, binned and essentially treated as junk mail:

‘Well, the first thing you would do with that [GP letter and reply slip] is bin it.’ (Male 64, Charleston)

Although there was some skepticism about the effectiveness of a letter from the GP in securing the desired response rates, participants appreciated the fact that a choice was given to reply and to express whether they would or would not be interested. They also appreciated that the letter encouraged people to think about the study after reading the leaflet, before making a decision:

‘I think [option] A is the best one out them all…. You know, send the slip back to say “Yes, you want to take part”, or if they’re not interested, don’t send it back, you know…’ (Male 64, Darnley)

‘It’s asking you to think about it as well, not just if you want to, it’s saying take your time and think. That’s nice.’ (Female 65, Castlemilk).

Nobody supported the idea of leaflets being handed out at the GP surgery (options C and D), either because they felt they were unlikely to visit the GP surgery (in order to be given a leaflet there) or that people would simply pay no attention to leaflets:

‘I don’t think they should give out leaflets, ‘cause so many people just put them in their pockets and that’s it… It’s a complete waste of time.’ (Female 54, Douglas)

Similarly, in both Glasgow and Dundee, respondents mentioned that they did not regularly visit their GP and so the consultation approach (option E) would not work:

‘It depends how often you see your GP. For example, the last time I saw my GP was when I got my hip replacement four years ago. Now, possibly, I’m lucky. But if I’m never near my GP, then that option isn’t gonna work for me, you know.’ (Male 66, Charleston)

‘Me either. I mean, it’s two years, or three years, and it was fourteen years before that before I was at my doctor.’ (Female 62, Charleston)

Only a minority of participants felt that a face-to-face invitation from the GP would be the best approach to take, because it was more direct and personal.

A small number of participants suggested that leaflets handed out whilst collecting repeat prescriptions may work:

‘I think the leaflet when you’re getting your repeat prescription. ‘Cause let’s face it, everybody goes for their repeat prescription.’ (Female 57, Castlemilk)

Focus group participants made an alternative suggestion to the GP invitation, which was word of mouth. Whilst the trial catchment areas were large, disadvantaged areas cluster in well-defined locations, and so word of the study was likely to spread amongst people in these areas. Different forms of media, especially local papers and the radio, were also seen as potentially useful for raising awareness about the study, which may make people more comfortable about taking part (giving the study a local public profile). However, the idea of study posters received mixed views. Some suggested they may be useful if they were posted in GP surgeries, hospitals or local community centers, but others questioned the impact they would have.

### Following up the study invitation

Views regarding the follow-up of non-responders were mixed. Although people generally acknowledged that the principle of follow-up was a good idea, especially to capture people who genuinely wanted to take part but who had forgotten to respond, people in all groups almost unanimously did not like the idea of follow-up telephone calls for non-responders (option B). The general perception was that follow-up calls from a researcher or receptionist would be viewed as akin to a sales and marketing call:

‘The phone…it’s always somebody trying to sell you something. You know, how many times in the day do you get that?’ (Male 75, Darnley)

The idea of follow-up reminders by text messaging was also dismissed. The most popular option to follow-up non-responders was being asked directly by GPs (or receptionists) during routine appointments:

‘Well, see when you go to your Doctor normally anyway. Well, how can he not be able to ask you if you want to participate?… *‘*Cause you’re there anyway.’ *(*Male 55, Douglas)

## Understanding randomization and issues related to the control group

### Understanding randomization

Some participants struggled to understand the concept or need for randomization:

‘How do they choose? Say, likes of five will go for the test and five will’nae, how do they actually choose?’ (Male 64, Darnley)

‘Is it an even, uneven numbers?’ (Female 65, Darnley)

‘Likes of, you know, if you’ve got somebody talking about their health problems, is that how it’s determined?’ (Male 64, Darnley)

However, these concerns were allayed by the facilitator who was able to explain the scientific rationale for, and process of random allocation. Participants reported feeling more inclined to take part once this was explained further, indicating the importance of explaining the need for randomization in an understandable way. Participants suggested ways of explaining randomization to their peers, using terms such as *half and half*, *picking names out of a hat* or *like the lottery* to reassure people that decisions about groups were not based on personal characteristics:

‘I mean, the term “randomization” is clear, but how is it done, is it through a computer or…I think I’d like to see, maybe if they’re gonna do it like that it says that they’re gonna pick it from the computer, names, you know, so people know that the reason why they’re not chosen is because of the computer. It’s like what Radio Clyde do, the computer picks the winner!’ (Male 64, Darnley)

‘Like the lottery. That’s something that everybody understands.’ (Female 71, Darnley)

Despite explaining random allocation, some participants were still uncertain whether they would be selected on the basis of some personal or illness characteristics:

‘I might be more worried if I was put in the test group, that they maybe thought there was something there… You’d think, “Oh my God, have they seen something?” I think you would.’ (Female 57, Castlemilk)

‘Well, if someone is in poor health, maybe they should have a better chance of being in the test group.’ (Female 65, Castlemilk)

This suggests that, for some, the randomness of the allocation to study groups might still be questioned. Furthermore, although participants perceived the random approach to be fair, most people expressed that they would clearly prefer to be in the test (experimental) group:

‘I think most people would want to be in the ‘top group’ (test group), but I think everybody is prepared to know that they are going to be half and half.’ (Female 54, Douglas)

### Issues related to the control group

Some participants struggled with understanding the rationale for having a control group, and said that allocation to the control arm of the study would put them off from participating:

‘Moderator: So, the fact that there’s a 50% chance that you wouldn’t get tested means that you wouldn’t want to take part?

Aye. If I was one of the 50% when they said, “Right, we’re gonna take a sample from you and test it”, then yeh, but if I was one of the 50% that didn’t get picked (the control group), then no. I would rather not know, actually. No.’ (Female 63, Charleston)

Some participants, however, understood this need and attempted to convey this to others in the group, as evidenced in the following exchange:

‘I think if they’re gonna do the study, they should do it to everybody (the test). The whole lot should have a chance to.’ (Female 57, Castlemilk)

‘But that’s, they’ve got to be able to compare it with people who have not been tested with it to see how effective it is.’ (Male 56, Castlemilk)

‘But say like, they take 50 people, well, can they not ask certain doctors to take part in and let the doctors do the other 50 who are not taking part in it?’ (Female 57, Castlemilk)

‘They’ve got to have something they can measure it against, it’s like a placebo. They’ve got to have something to check against it.’ (Male 56, Castlemilk)

However, even for some people who understood the need for a control group, they found it hard to appreciate the need for this in a screening trial*:*

‘You can understand if it was a medication that was coming out and it was a blind trial, they need people to take the placebo and other people not, but you would think it’s different with this kind of thing.’ (Male 64, Charleston)

Participants were asked about the label usual care group to describe the control group. Some felt that the label was not clear:

‘I wasn’t very sure of it when I read it at first. I had to think about it, “What’s the usual care group?” You have to read through it before you realize what it is.’ (Female 69, Douglas)

‘It’s not really anything is it?’ (Female 69, Douglas)

‘I was gonna ask, “What is that?” but I thought, “No, I’ll read on a bit”.’(Female 69, Douglas)

Others thought the term was misleading:

‘It makes it sound like you’re gonna get some kind of care, but you’re not getting any kind of care.’ (Female 54, Douglas)

‘I don’t think that’s the right title for it.’ (Male 55, Douglas)

Suggestions for alternative names for the usual care group included: research care group, research group, untested group, placebo, and non-test group.

Although the idea that people would be allocated at random to one of the two groups did not appear to attract any confusion among participants, this was the one area of the study that did cause some to question whether or not they would consider taking part in the main trial:

‘So, if I was told that I wasn’t going to be in the test group, I would say, “Well, what’s the point in doing it then?”’ (Female 71, Darnley)

‘It wouldn’t bother me, but it might bother a lot of people – “Well, you’re not getting my blood then. If I’m not gonna be in the test group, you’re not taking my blood”.’ (Female 54, Douglas)

A view that was asserted in all groups was that the blood test should be offered to all participants, and not just half of them. Comments from some participants demonstrated a lack of understanding of the scientific nature of the study and the need for a control or comparison group:

‘Would they not be better, instead of having half of the people not going through the full trial, could they not put everyone in the full trial?… Surely, it must be more beneficial to test 10,000 people to see if they’ve got lung cancer than just 5,000 people in the trial?’ (Male 64, Charleston)

‘But, how are they only doing 50%? Why do they not just test them all?’ (Male 63, Castlemilk)

Clearer explanations of the rationale for randomization, and how and when people would be informed of their group allocation were therefore needed.

## Perceived barriers and facilitators to participation

We class the perceived barriers as practical barriers and psychosocial barriers. Of the former, the main obstacle to participation appeared to be the need for flexible appointments that were local to participants. Reimbursement of travel expenses was not considered important to most as they had free bus passes. Among those who did not qualify for bus passes because of their age, one said that they would welcome the reimbursement and one did not. Reimbursement of travel expenses was only seen as necessary if appointments were further than the local GP surgeries (at a hospital or elsewhere further from home).

While most of the respondents were retired, work commitments among some of the younger participants were seen as a potential barrier and so the need for flexible appointments was perceived to be greatest for this demographic (under 60s):

‘Well, your appointments would have to be flexible, because people are still working. Not myself, I’m retired, but there are always people working who might not be able to get time off work.’ (Male 64, Charleston)

With regard to perceived psychosocial barriers, participants felt stigmatized (because of their smoking status) by some of the language used in the PILs (such as targeting smokers, because of their higher risk of developing lung cancer). Some strong views were expressed that cancer could affect anyone and smokers should not be made to feel singled out or challenged:

‘You’re saying smokers, but there are people that have got cancer that have never smoked in their life…’ (Female 63, Charleston)

‘There’s folk that haven’t smoked in their life. Folk sitting in a pub, or sitting in a hall and catch it [cancer].’ (Male 75, Douglas)

One possible barrier to recruitment was the perception held by some participants that the trial is designed to encourage people to stop smoking. The following exchange between the moderator and one participant highlights this view:

‘Moderator: It’s [the PIL] preaching a wee bit?

Participant: Aye, “You smoke. Please stop ‘cause it’s gonna give you lung cancer.”

Moderator: Is that the message that you get, that it is saying that you should stop (smoking)?

Participant: Yeah.

Moderator: ‘Cause, um, I should have said, if you took part in the study no one would ask you to stop smoking.

Participant: It was the same as when I was stopped in the street (for recruitment), they were asking me if I smoke, and how many I smoke, and you go, what do you say? “Are you trying to get me to stop smoking?” eh?’ (Female 63, Charleston)

This was evident in another exchange between the moderator and a participant:

‘Participant: Would you want the person to stop smoking if they took part in that?

Moderator: No.

Participant: It’s just that there’s lots of questions about your smoking.’ (Female 57, Castlemilk)

Indeed, some participants believed that by taking part in the trial and finding out that they had lung cancer may force people to stop smoking, even though they did not want to:

‘Giving up smoking… I’m just saying that if you’re told you’ve got lung cancer, you’d stop smoking today, you know what I mean? I think that would be hard for a lot of people. Even if they said you’d got small lung cancer…’ (Female 54, Douglas)

Such views were, however, contrasted with equal numbers of participants who said that they would continue to smoke, regardless of what the test found:

‘I don’t agree with that. If I find out I’ve got cancer, there’s no way I’m gonna stop smoking.’ (Female 57, Castlemilk)

‘“Are you gonna give up smoking?” ’ Not a chance! (Female 60, Darnley)

In general, participants felt that those who were willing to participate would be committed to the trial for its duration unless appointment times were too rigid, there were too many appointments or appointments were too far away, or if they became unwell during the study:

‘You’re either gonna take part or you’re not, once you’ve got all the information, and once you’ve made your choice, that’s it.’ (Male 56, Castlemilk)

Altruism was perceived to be a motivator for participants, particularly for those in the control group who saw their role in participating in the research, even to the extent that they viewed the blood they were giving (which was not going to be tested for the lung cancer) as helping others:

‘The way I look at it is that I can’t do much for ‘mankind’, but if giving a wee vial of blood helps…. I think that should be explained then when they go to the nurse at the beginning when you’re told that not in the test group, but you will be helping your fellow man, or whatever you want to put it, you will be helping. You will be contributing to the research.’ (Female 71, Darnley)

‘We’ve all got children, grandchildren, families, so if it’s gonna help them eventually, then. ‘Cause it’s a disease isn’t it? Lung cancer.’ (Female 60, Darnley)

‘Every little helps. Even if you’re helping somebody else.’ (Male 56, Castlemilk)

‘But it is for medical research, it’s for the benefit of others, so.’ (Female 54, Douglas)

## Discussion

We conducted four focus groups to elicit views about various aspects of the ECLS trial amongst members of the public who matched the inclusion/exclusion criteria for the trial. We felt this would enable us to refine our trial procedures and participant facing documentation to enhance recruitment in potentially hard to reach groups. As Shaghaghi, Bhopal, and Sheikh [28] concluded in their review of literature on recruiting hard to reach groups, recruitment will depend on the characteristics of the group, recruitment techniques used, and the subject of interest. Our study enabled us to get a better understanding of the characteristics of this group. We found that a combination of approaches would be needed to meet the recruitment preferences of those who took part in the focus group sessions. We also found wide differences between focus group members in terms of the regularity with which they saw their GP, their likelihood of collecting regular repeat prescriptions, and responding to postal invitations. That said, overall the invitation letter from the GP was the most popular of all recruitment options presented, followed up either by a reminder letter or a face-to-face reminder when participants next visited their GP (see Table 2 for a list of changes made to the final study design and PIL based on the feedback from the focus groups). The apparent contradiction between the perceived usefulness of face-to-face invites and face-to-face reminders from GP may reflect participants’ experience, where non-attenders at national screening programs may be followed up by their GP, as recommended in guides to maximize screening attendance [29].

**Table 2:** Themes, issues raised and changes made

**Theme**	**Issues raised**	**Changes made to the study design or Participant Information Leaflet (PIL) to address the issues**
Recruitment options: How to approach potential participants	Support for study information to be sent by GPs or to be given in consultations	Recruitment therefore comprised:
	Support for having a summary of the main points in the PIL	a. Postal invitation letter including the study PIL with a summary of the main points at the front of the PIL;
	Lack of support for receptionists or researchers handing out study information leaflets at the practice	and, where necessary or appropriate:
	Participant suggestions that attaching PIL to repeat prescriptions may be helpful	b. Invitation letter including a summary of the study PIL on collection of repeat prescription;
	Concern that those who don’t regularly go to the GP would not be reached and need for other approaches	c. Invitation during consultation with GP/Practice Nurse/Health Care Assistant at the practice;
	Importance of hearing about study through “word of mouth” and using the media to publicize study	d. Invitation to those eligible on registered research volunteer databases
		e. Posters in GP waiting rooms;
		f. Other recruitment strategies including; Media campaign involving: local and national newspapers; BBC Scotland; local radio, celebrity endorsement
		g. Publicity campaign using posters/leaflets etc. at venues such as:
		Football/Bingo halls/Bowling clubs; Smoking Cessation Clinics; Hospital main entrances/hospital clinics; Shopping Centers/Supermarkets/Pubs, etc.; Benefits offices/Post offices, etc.; Sheltered Housing /Housing Associations
		h. Community and charitable outreach programs, mobile screening clinic, pharmacist approach through practices.
Recruitment options: How to follow up non-responders	Acceptance of need to follow up non responders to give people who genuinely wanted to take part but who had forgotten to respond the opportunity to do so	Study invite reply slip gives patients the option to specify they do not want to be contacted again by research team. Non-responders are followed up by mail with one reminder. Practices will be offered the option of using the Trial Torrent software package to identify potentially eligible patients who do not respond to postal study invites so that they can be approached during routine primary care consultations.
	Dislike of telephoning or texting non-responders	
	Support for non-responders being asked by GP or nurse during consultations	
Practical barrier: Appointment times	Participants felt work commitments could be a barrier to participation and flexible appointment times would be needed	Those expressing interest in the study are sent the full PIL and at least 24 hours after anticipated receipt are phoned to discuss the study, answer questions, undertake a preliminary eligibility assessment and to arrange a recruitment visit at a time suitable to the patient. Patients are reminded of the appointment by phone, text message or email 48 hours prior to the appointment depending on their preferred method of contact.
Psychosocial barrier: Stigma	Participants felt stigmatized about being targeted because of where they lived.	‘These [geographical] areas have been chosen because we know that lung cancer is more common in these areas.’*
	Participants felt targeted for being smokers and wanted the researchers to acknowledge that lung cancer can also occur in non-smokers.	‘Lung cancer can happen to anyone, including the young and old and people who do not smoke, but the risk is higher in those over 50 and those who have smoked.’
	Participants were concerned that by taking part in the trial, researchers would encourage them to stop smoking.	We removed all mention of providing smoking cessation information and advice from the PIL.
Issues related to the control group	Participants did not like the use of the term *usual care group* to describe the control group	We changed this to *non-test group*, which is what participants were most comfortable with.
	Participants did not always understand why a control group was needed in the trial.	‘Whenever a new test is developed we need to find out if it works. We do this by having a group of people who have the test and a group of people who do not. Both groups need to be similar so that we can compare what happens to the people in each group.’
	Some participants said they would be put off from participating if they were assigned to the control group	‘If you are in the non-test group, the information you give us will be really important in helping us find out if the new lung cancer blood test works, by comparing what happens to both groups. By taking part, you are playing an essential role in the research and could be helping future generations.’
		‘People in the non-test group are still playing a very valuable role in the research by allowing a comparison with those whose blood is tested, and could also be helping future generations – ECLS Team’
Not understanding randomization	Participants feared that they would be chosen for some specific personal characteristic identified by someone in the trial or their doctors	‘To try and make sure both groups are the same, each person is put into a group at random. This is done by a computer putting people into one of the two groups by chance. This is the fairest way of deciding who gets the test and means everyone will have a 50/50 chance of being put in either group. This means that you are not chosen to be in a group for any particular reason.’
Understanding the nature and purpose of the test	Some participants thought the test would pick up other health problems.	‘The test is only looking for lung cancer, so will not pick up other types of cancer or other diseases.’

* Statements in quotes reflect the final wording used in PIL.

The themes, based on the focus group data, highlighted mixed understanding and mixed feelings about various concepts associated with RCTs. Based on these (mis)understandings, potential participants may not have taken part in the main trial. Participants grappled with the concept of randomization and the need for a control group, even with a facilitator being present to explain these concepts. Not understanding the need for a control group may deter participation and increase control arm dropout rates. This highlights difficulties likely to be faced by trials that first approach potential participants with a postal invitation letter and/or PIL. Our participants did suggest alternative explanations, wordings and labels for randomization and the control group, which they thought would make the study clearer for potential participants.

When participants understood these concepts, the most common reason for agreeing to participate, in the absence of any personal gain, appeared to be altruism. Within reason, most participants felt that even if they were allocated to the control arm they would be happy for their blood to be collected and to be used for other medical research. The importance of altruism as a motivator for trial participation is consistent with the findings of the systematic review by Ross *et al.*[5].

Participants articulated some barriers that would prevent them from participating in the main trial, especially practical ones such as too few and lack of flexible appointment options, and attendance to research sites that were not local to them. Some participants, however, felt singled out or stigmatized because of their smoking behavior, and wanted the PIL to be clear that it was not only smokers who developed lung cancer. There was also some concern that the trial, if seen to be promoting smoking cessation, would deter people from participating. These perceived psychosocial barriers prompted us to carefully reconsider, and make changes to all of our participant facing material. It is important to note that, while the recruitment sought to achieve a broad mix of demographics across a range of geographical areas, the findings presented here cannot be considered as representative of the communities from which the participants were drawn, and instead only provide indicative insight into the target population’s views. We acknowledge that participant factors are only one aspect of recruitment, and that even with interest from potential participants, removal of other institutional barriers (such as those identified by Patterson *et al.*[30]) is essential for adequate recruitment.

## Conclusions

Focus groups provided us with useful information which resulted in numerous changes to proposed trial documentation and processes in order to better address participant’s information needs. Based on our findings, we altered the language to reduce misunderstanding regarding randomization and the control group, and to ensure that smokers did not feel singled out for developing cancer. This was achieved in the following ways: we removed mention of smoking cessation from our PIL; we decided on the best ways to invite potential participants to take part; and were reassured that our questionnaires (particularly those related to smoking behavior) would not alienate participants. The results from our focus groups may be transferable to other community based screening trials recruiting a similar participant pool. However, we would recommend that trials, or feasibility studies for trials budget for and conduct their own pretrial focus groups to better recognize the issues that may influence participants’ willingness to engage with the trial. In the long run, this may be cost effective for the trial.

While some interventions to improve recruitment in primary care trials have been assessed [31], and the use of user testing to improve readability and understanding of trial information for participants has been evaluated [10], the effectiveness of pretrial engagement with a subgroup of potential participants in improving participant facing material to improve recruitment has not yet been systematically evaluated. Further research is required in this area.

## Appendix

### Focus group topic guide

1. Brief introduction (2 minutes)

**2. Aims of the FG:***Explain the reason why they have been invited to take part and the aims of this session***(3 minutes)**

**3. Summary Section from the Participant Information Sheet:***Go through the Summary section and see whether this is clear***(5 minutes)**

–Having read this summary sheet, how well do you think it explains what the study is about?

–Does the summary sheet raise any questions or concerns for you?

what are they?

would they affect your willingness to take part?

would they affect your willingness to read on and find out more about the study?

Do you think this summary sheet is needed?

4. General questions about the study (5 minutes)

–Would the blood test be something you would think about having?

–What kind of issues/concerns/worries would you have about having such a blood test?

–Do you think there might be benefits of having such a test? What might these be?

–Do you think there might be disadvantages to having such a test? What might these be?

–With the information you have received so far, how likely is it that you would take part in the study if you were invited to? (ask all for a separate response)

**5. Recruitment:***Explain who we would like to recruit (inclusion criteria)***(15 minutes)**

–We are thinking about several different ways to tell people about the study and ask them if they might be interested in taking part. At the moment we are thinking about the following: **[Use SHOW CARD 1] Stress that 90% of GPs in the area have already agreed to take part.**

–What do you think about these ways of getting people involved in the study?

–Specifically, how would you feel about the letter being followed-up with a phone call if you didn’t respond? Would this bother you?

–What do you think of:

Radio? (which channels?)

Newspapers? (which ones?)

Word of mouth? (how would this work?)

Posters? (where?)

–Are there any other ways of asking people if they want to take part that you think might work?

–What do you think of this letter? (Show example GP letter)

–If it was you, how would you like to be invited to take part in a study of this kind? (either choose from the options listed or ‘other’) Take count.

6. GP Letter and Participant Information Leaflet: (20 minutes)

–What do you think of this information leaflet?

–Is the language clear?

–Are there any words that are difficult to understand?

–Does it give you enough information to help you decide whether you want to take part in the study?

–Is there anything missing that you would have liked to have seen in the letter and leaflet?

–Is there too much information in the letter and information leaflet?

–Is there anything that could be left out? If so, what?

–How clear is it about the advantages and disadvantages (including the risks) of taking part in the study? Please explain this in your own words, as if explaining it to someone else.

–Do you understand what it would mean if the test was positive? What would it mean if the test was negative? Please explain this in your own words, as if explaining it to someone else.

–Do you understand what a false positive test is? Do you understand what a false negative test is? Please explain this in your own words, as if explaining it to someone else.

–Would you be happy to receive this information through the post; or would it be better to receive the summary and be able to go through the rest of the information with a nurse?

–What do you think of this kind of leaflet? **(use Tascforce leaflet as example)**

–What do you think of this kind of diagram, as a way of explaining the study? **(Use Flowchart)**

–If you were trying to show someone what will happen in the study, how would you do it? (using images rather than words)

### BREAK (10 minutes)

**7. Randomisation:***Explain what this entails (including clinical equipoise (*i.e. *we really do not know whether the test will save lives or what the advantages and disadvantages of having the test are for people) and terms such as ‘lung cancer test group’ and ‘usual care group’).***Use SHOW CARD 2 (20 minutes)**

–How well does the information leaflet explain how people will be chosen for the lung cancer test group and the usual care group? (i.e. at random)

–How would you explain this to someone, in your own words?

–Could we explain this any better? If so, how?

–What do you think about the way that people are put into the two groups? (i.e. at random)

–How would you feel if you were told you had an equal chance of being in the ‘lung cancer test group’ or the ‘usual care group’?

–If you were in the ‘lung cancer test group’, how would that make you feel?

–If you were in the ‘usual care group’, how would that make you feel? Would you still consider going through to the end of the study?

–We have been talking about the control or usual care group having their blood taken and it being used for medical research. We would like to know how you feel about having your blood taken if you are in the control or usual care group? (Prompts- might this put some people off taking part in the study? Would some of you only think about taking part if the control or usual care group didn’t have any blood taken at all?)

–Even if your blood was not going to be tested as part of this study, would you still be happy for it to be used by other researchers?

–What do you think of the label ‘usual care group’? Does this sound negative? If so, why?

–It is important that people realise that, by being in this group, they will still be making an important contribution to the study. Is there another name that you think would be better for this group?

8. Consent form: (5 minutes)

–What do you think about this consent form?

–Is it clear what you are agreeing/consenting to?

–Is the language clear? Are there any words which are difficult to understand?

–Is there anything that needs adding or clarifying?

9. Questionnaires: (15 minutes)

–Are you happy with the number of questions you have to answer?

–Are there any of the questions about smoking that you feel are inappropriate and/or you would not be willing to answer and why?

–Do you have a preference (and if so why) on where in the booklet these smoking questions are asked?

–[Re: the cancer worry scale] Are there any items you do not like or do not understand? If so, why?

–[Re: the PANAS] Do you understand why we are asking these questions? Please can you explain in your own words?

–Questionnaires may take 10–15 minutes to complete. How do you feel about that?

–Would you have a preference for when you would receive this questionnaire? For example, before your meeting with the nurse, or at the meeting?

–How would you like to respond to the questions?

•Answer them yourself?

•Have someone read out the questions and you respond verbally, so that they can fill out the questionnaires for you?

–Would you mind receiving the questionnaires in the post, filling them out, and sending them back in a pre-paid envelope?

–Would you mind completing the questionnaire over the telephone?

–Are there any other ways you would prefer to complete these questions (e.g., computer/web)?

–How would you feel about receiving £5 for every questionnaire booklet you completed over the study?

•Would it make you more or less likely to take part?

•Is £5 enough?

10. What will help people remain in the study and prevent them from leaving prematurely? (15 minutes)

–If you thought that you might like to take part, is there anything that might make it easier for you to take part? (For example, reminders about appointments, payment for childcare, travel expenses, flexible appointment times, duration of the study, the number of times you need to be assessed, etc.)

–What could we do to make it easier for people to take part in this study?

–If you joined the study, what might make it difficult for you to stay in the study? (For example, summer holidays, school breaks, travel to appointments, getting time off work, too many questionnaires to fill in (how many is too many?), etc.)

–What might make you want to stop taking part?

How likely are you to take part in the study? (5 minutes)

–Now that you have found out more about the study, if you were invited to take part, how likely is it that you would take part? (ask all for a separate response)

–If you have changed your mind from when we asked you at the beginning of the group, what made you change your mind?

Thank and close

## Abbreviations

ECLS: Early Cancer detection test – Lung cancer Scotland; GP: General Practitioner; MRC: Medical Research Council; PIL: Participant Information Leaflet; RCT: Randomized Controlled Trials; UK: United Kingdom.

## Competing interests

RdN, KV, & DK are involved in the ECLS trial. The authors declare no other competing interests.

## Authors’ contributions

RdN was involved in conception and design, secured funding and ethics approval, data analysis, manuscript writing and final approval of the manuscript. KSO was involved in data collection and analysis, critical revision and final approval of the manuscript. KV was involved in conception and design, secured funding and ethics approval, data analysis, critical revision and final approval of the manuscript. DK was involved in conception and design, secured funding and ethics approval, data analysis, critical revision and final approval of the manuscript. All authors read and approved the final manuscript.

## References

[B1] Campbell MK Snowdon C Francis D Elbourne D McDonald AM Knight R Entwistle V Garcia J Roberts I Grant A the STEPS group Recruitment to randomised trials: strategies for trial enrolment and participation study. The STEPS study Health Technol Assess 2007 11 iii, ix-105 17999843 10.3310/hta11480

[B2] Bower P Wilson S Mathers N How often do UK primary care trials face recruitment delays? Fam Pract 2007 24 601 603 10.1093/fampra/cmm051 17872907

[B3] Tognoni G Alli C Azanzini F Bettelli G Colombo F Corso R Marchioli R Zussino A Randomised clinical trials in general practice: lessons from a failure BMJ 1991 303 969 971 10.1136/bmj.303.6808.969 1954424 PMC1671349

[B4] Altman DG Bland JM Absence of evidence is not evidence of absence BMJ 1995 311 485 10.1136/bmj.311.7003.485 7647644 PMC2550545

[B5] Ross S Grant A Counsell C Gillespie W Russell I Prescott R Barriers to participation in randomised controlled trials: a systematic review J Clin Epidemiol 1999 52 1143 1156 10.1016/S0895-4356(99)00141-9 10580777

[B6] Fayter D McDaid C Eastwood A A systematic review highlights threats to validity in studies of barriers to cancer trial participation J Clin Epidemiol 2007 60 990.e1 990.e33 17884592 10.1016/j.jclinepi.2006.12.013

[B7] Weller DP Campbell C Uptake in cancer screening programmes: a priority in cancer control B J Cancer 2009 101 s55 s59 10.1038/sj.bjc.6605391 PMC2790712 19956164

[B8] Baron RC Rimer BK Breslow RA Coates RJ Kerner J Melillo S Habarta N Kalra GP Chattopadhyay S Wilson KM Lee NC Mullen PD Coughlin SS Briss PA and the Task Force on Community Preventive Services Client-directed interventions to increase community demand for breast, cervical, and colorectal cancer screening: a systematic review Am J Prev Med 2008 35 S34 S55 10.1016/j.amepre.2008.04.002 18541187

[B9] Treweek S Pitkethly M Cook J Kjeldstrøm M Taskila T Johansen M Sullivan F Wilson S Jackson C Jones R Mitchell E Strategies to improve recruitment to randomised controlled trials Cochrane Database Syst Rev 2010 14 MR000013. doi:10.1002/14651858.MR000013.pub5 10.1002/14651858.MR000013.pub5 20393971

[B10] Knapp P Raynor DK Silcock J Parkinson B Can user testing of a clinical trial patient information sheet make it fit-for-purpose? - A randomized controlled trial BMC Med 2011 9 89 10.1186/1741-7015-9-89 21777435 PMC3152894

[B11] Robinson EJ Kerr CE Stevens AJ Lilford RJ Braunholtz DA Edwards SJ Beck SR Rowley MG Lay public understands of equipoise and randomisation in randomised controlled trials Health Technol Assess 2005 9 1 192 iii-iv 15763039 10.3310/hta9080

[B12] Featherstone K Donovan JL Random allocation or allocation at random? Patients’ perspectives of participation in a randomised controlled trial BMJ 1998 317 1177 1180 10.1136/bmj.317.7167.1177 9794849 PMC28698

[B13] Edwards SJL Lilford RJ Braunholtz DA Jackson JC Hewison J Thornton J Ethical issues in the design and conduct of randomised controlled trials Health Technol Assessment 1998 2 i-vi 1 132 10194615

[B14] Williams B Entwistle V Haddow G Wells M Promoting research participation: why not advertise altruism? Soc Sci Med 2008 66 1451 1456 10.1016/j.socscimed.2007.12.013 18222579

[B15] Allin S Masseria C Mossialos E Inequality in health care use among older people in the United Kingdom: an analysis of panel data LSE health Working Paper No. 1/2006 2006 London: School of Economics and Political Science [http://eprints.lse.ac.uk/19262/1/LSEHWP1.pdf]

[B16] Rapport F Storey M Porter A Snooks H Jones K Peconi J Sanchez A Siebert S Thorne K Clement C Russell I Qualitative research within trials: developing a standard operating procedure for a clinical trials unit Trials 2013 14 54 10.1186/1745-6215-14-54 23433341 PMC3599333

[B17] Donovan J Hamdy F Neal D Peters T Oliver S Brindle L Jewell D Powell P Gillatt D Dedman D Mills N Smith M Noble S Lane A Prostate testing for cancer and treatment (ProtecT) feasibility study Health Technol Assess 2003 7 1 88 12709289 10.3310/hta7140

[B18] Craig P Dieppe P Macintyre S Michie S Nazareth I Petticrew M Developing and evaluating complex interventions: the new Medical Research Council guidance BMJ 2008 337 a1655 10.1136/bmj.a1655 18824488 PMC2769032

[B19] Gibbs A Focus Groups Social Res Update 1997 19 (Winter). [http://sru.soc.surrey.ac.uk/SRU19.html]

[B20] Fereday J Muir-Cochrane E Demonstrating rigor using thematic analysis: a hybrid approach of inductive and deductive coding and theme development Int J Quali Methods 2006 5 80 92

[B21] O’Reilly M Parker N ‘Unsatisfactory Saturation’: a critical exploration of the notion of saturated sample sizes in qualitative research Qual Res 2013 13 190 197 10.1177/1468794112446106

[B22] Green J Thorogood N Qualitative Methods for Health Research 2004 London: Sage

[B23] Spencer L Ritchie J Lewis J Dillon L Quality in Qualitative Evaluation: A Framework for Assessing Research Evidence 2003 London: Government Chief Social Researcher’s Office, Prime Minister’s Strategy Unit http://www.civilservice.gov.uk/wp-content/uploads/2011/09/a_quality_framework_tcm6-38740.pdf

[B24] Dey I Grounding Grounded Theory: Guidelines for Qualitative Inquiry 1999 San Diego, CA: Academic

[B25] Morse J Denzin NK, Lincoln YS Designing Funded Qualitative Research Handbook of Qualitative Research 1994 Thousand Oaks, CA: Sage Publications 220 235

[B26] Angen MJ Evaluating interpretive inquiry: reviewing the validity debate and opening the dialogue Quali Health Res 2000 10 378 395 10.1177/104973230001000308 10947483

[B27] Sandelowski M Rigor or rigor mortis: the problem of rigor in qualitative research revisited Adv Nurs Sci 1993 16 1 8 10.1097/00012272-199312000-00002 8311428

[B28] Shaghagahi A Bhopal RS Sheikh A Approaches to recruiting ‘hard-to-reach’ populations into research: a review of the literature Health Promo Perspect 2011 1 86 94 10.5681/hpp.2011.009 PMC3963617 24688904

[B29] Zelenyanszki C Maximizing screening attendance: a reference guide North West London Cancer Networkhttp://www.screening.nhs.uk/getdata.php?id=14467

[B30] Patterson S Kramo K Soteriou T Crawford MJ The great divide: a qualitative investigation of factors influencing researcher access to potential randomised controlled trial participants in mental health settings J Men Health 2010 19 532 541 10.3109/09638237.2010.520367 21121823

[B31] Foy R Parry J Duggan A Delaney B Wilson S den Broek NTHL-v Lassen A Vickers L Myres P How evidence based are recruitment strategies to randomized controlled trials in primary care? Experience from seven studies Fam Pract 2003 20 83 92 doi:10.1093/fampra/20.1.83 10.1093/fampra/20.1.83 12509377

